# Precision immunomodulation for pediatric hemophagocytic lymphohistiocytosis in intensive care

**DOI:** 10.1002/ped4.70087

**Published:** 2026-09-15

**Authors:** Weerapong Lilitwat, Prakreeti Bhandari

**Affiliations:** ^1^ Department of Pediatrics Texas Tech University Health Sciences Center Lubbock Texas USA

**Keywords:** Anakinra, Cytokine release syndrome, Hemophagocytic lymphohistiocytosis, Macrophage activation syndrome, Ruxolitinib

## Abstract

Hemophagocytic lymphohistiocytosis (HLH) and related cytokine storm syndromes are life‐threatening hyperinflammatory disorders that frequently intersect with pediatric critical care. Critically ill children may present with fever, shock, cytopenias, liver dysfunction, coagulopathy, hyperferritinemia, respiratory failure, neurologic deterioration, and multiple organ dysfunction, creating substantial overlap with severe sepsis. Traditional diagnostic criteria remain foundational but do not identify the dominant mechanism or optimal therapy for an individual child. In this review, precision immunomodulation is defined operationally as matching the suspected trigger and clinical phenotype with pathway‐informed biomarkers when available, drug‐delivery feasibility during organ failure, and the plan for definitive disease control. Interferon‐gamma activity may be supported by CXCL9; interleukin (IL)‐1/IL‐18 biology may support anakinra in macrophage activation syndrome; broader cytokine signaling may support Janus kinase inhibition in selected refractory HLH; and IL‐6‐directed therapy is primarily syndrome‐ and grade‐directed in immune effector cell cytokine release syndrome. These markers are supportive rather than validated treatment thresholds. Targeted therapy must proceed alongside antimicrobial therapy, source control, organ support, infection surveillance, and early hematopoietic stem cell transplantation planning when indicated. This review provides a bedside framework for phenotype assignment, biomarker interpretation, intensive care unit‐focused targeted therapy, extracorporeal bridge strategies, and reassessment at 24–72 h.

## Graphical abstract

This review presents a bedside framework for recognizing pediatric hemophagocytic lymphohistiocytosis and cytokine storm, stabilizing organ dysfunction, identifying the underlying phenotype, selecting targeted immunomodulation, considering extracorporeal adjuncts, and reassessing response within 24–72 h.

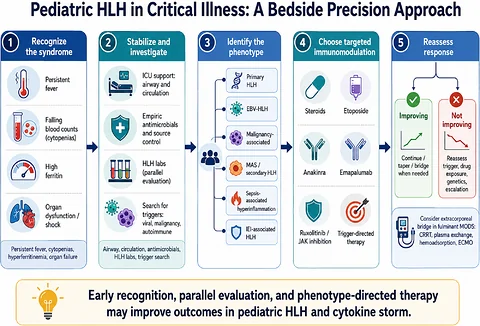

## INTRODUCTION

Hemophagocytic lymphohistiocytosis (HLH) and related cytokine storm syndromes are among the most challenging hyperinflammatory emergencies encountered in pediatric critical care. Affected children may present with persistent fever, shock, cytopenias, liver injury, coagulopathy, hyperferritinemia, respiratory failure, renal dysfunction, or neurologic deterioration. These findings often overlap with severe sepsis and multiple organ dysfunction syndrome, making early recognition difficult. Current critical care consensus guidance recommends prompt HLH testing in patients in the intensive care unit (ICU) with unexplained or disproportionate inflammation, particularly when rapid clinical deterioration is present.^1^

This diagnostic overlap is clinically consequential. A child with uncontrolled infection may be harmed by excessive immunosuppression, yet a child with untreated HLH may deteriorate despite appropriate antimicrobial therapy, vasoactive support, and organ support. The HLH‐2004 diagnostic criteria remain foundational in pediatrics, but they do not define the mechanism, trigger, or optimal therapy for each critically ill child.^2^ Practical critical care reviews emphasize that HLH may mimic or coexist with sepsis and should be suspected in an unwell febrile patient with falling cell counts and high ferritin concentrations.^3^ Pediatric studies comparing HLH and severe sepsis further demonstrate substantial overlap in organ dysfunction patterns.^4^

The traditional distinction between primary and secondary HLH remains useful but is increasingly insufficient. Primary or familial HLH is classically associated with inherited defects in cytotoxic lymphocyte function and often requires hematopoietic stem cell transplantation (HSCT) for cure. Secondary HLH may be triggered by infection, malignancy, autoimmune or autoinflammatory disease, immune effector cell therapy, or other immune stressors. In practice, these categories overlap. Infection may trigger familial HLH, and apparently secondary HLH may reveal an unrecognized inborn error of immunity (IEI). Genetic and mechanistic studies support conceptualizing HLH as a critical illness phenotype driven by toxic immune activation via diverse mechanisms rather than as a single disease.^5^

For the pediatric intensivist, the central challenge is to integrate phenotype‐directed immunomodulation with time‐sensitive organ support. Corticosteroids and etoposide remain important, particularly in familial HLH and severe, persistent, or relapsing disease. However, targeted agents are increasingly used in refractory or pathway‐defined phenotypes. Emapalumab targets interferon‐gamma, ruxolitinib and other Janus kinase (JAK) inhibitors interrupt multiple cytokine‐signaling pathways, and anakinra provides interleukin (IL)‐1 blockade, especially relevant to macrophage activation syndrome (MAS) and secondary HLH‐like cytokine storm. Extracorporeal approaches such as plasma exchange or hemoadsorption may have adjunctive roles in selected patients with fulminant hyperinflammation and multiorgan failure, but they remain bridge strategies rather than definitive therapies.

**FIGURE 1:**
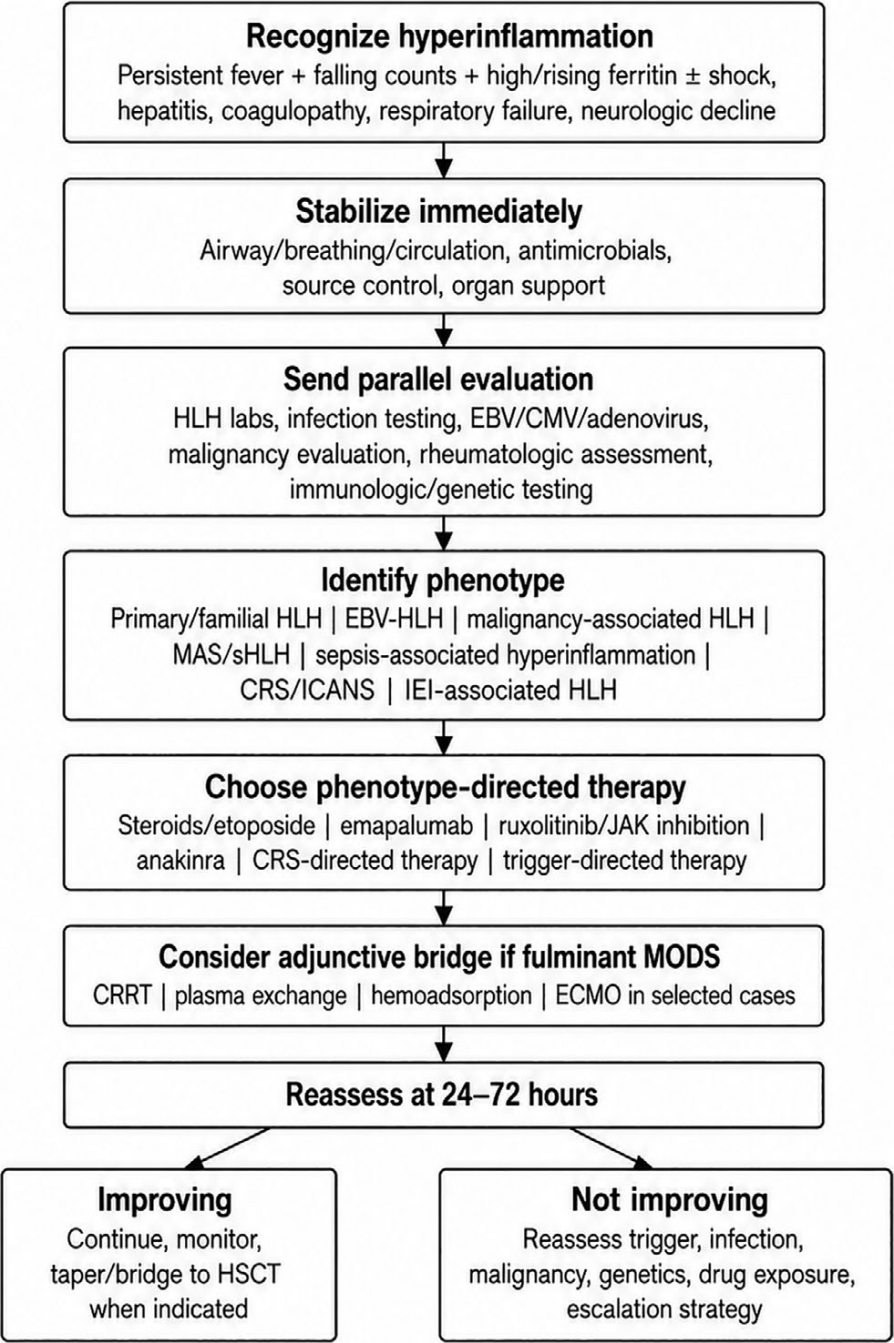
Bedside framework for precision immunomodulation in pediatric hemophagocytic lymphohistiocytosis and cytokine storm. The algorithm emphasizes simultaneous stabilization, diagnostic evaluation, trigger identification, and early multidisciplinary treatment. Hemophagocytic lymphohistiocytosis and cytokine storm may mimic or coexist with sepsis; therefore, antimicrobial therapy, source control, and organ support should proceed in parallel with hemophagocytic lymphohistiocytosis‐directed evaluation. When available, pathway‐informed markers such as CXCL9/interferon‐gamma activity, IL‐18 in MAS‐like biology, and serial EBV DNA may support phenotype assignment, but treatment should not be delayed while awaiting specialized cytokine testing. Immunomodulatory therapy should be selected according to phenotype, disease severity, organ‐failure context, infection risk, drug‐delivery feasibility, and the definitive disease‐control plan. Lack of improvement within 24–72 h should prompt reassessment for uncontrolled infection, occult malignancy, inborn error of immunity, inadequate drug exposure, incorrect inflammatory target, or need for escalation to targeted therapy, cytotoxic therapy, extracorporeal support, or hematopoietic stem cell transplantation planning. CMV, cytomegalovirus; CRS, cytokine release syndrome; CRRT, continuous renal replacement therapy; EBV, Epstein‐Barr virus; ECMO, extracorporeal membrane oxygenation; HLH, hemophagocytic lymphohistiocytosis; HSCT, hematopoietic stem cell transplantation; ICANS, immune effector cell‐associated neurotoxicity syndrome; IEI, inborn error of immunity; IL, interleukin; MAS, macrophage activation syndrome; MODS, multiple organ dysfunction syndrome; sHLH, secondary hemophagocytic lymphohistiocytosis.

This review focuses on bedside precision immunomodulation in critically ill children with refractory HLH and cytokine storm. Here, “precision” does not mean waiting for a comprehensive cytokine panel. It means integrating four decisions in parallel: (1) identify the most likely trigger and clinical phenotype; (2) use pathway‐informed biomarkers when available, such as CXCL9 as a surrogate for interferon‐gamma activity, IL‐18 in MAS‐like biology, and Epstein‐Barr virus (EBV) DNA burden and infected‐cell lineage in EBV‐HLH; (3) select an agent that can be delivered safely in the child's current organ‐failure state; and (4) define the definitive disease‐control plan, including HSCT when indicated. Biomarkers support, but do not replace, clinical phenotype assignment, and no validated numeric cytokine threshold should be used to delay treatment in an unstable child. Figure 1 summarizes simultaneous stabilization, diagnostic evaluation, phenotype identification, immunomodulatory selection, adjunctive support, and early reassessment. This is a narrative review; relevant English‐language literature was identified through PubMed using search terms including HLH, MAS, cytokine storm, biomarkers, and the individual targeted therapies, with emphasis on pediatric and critical care studies published through 2026 and selected adult or mechanistic references where pediatric evidence was limited.

## HLH AND CYTOKINE STORM AS PEDIATRIC CRITICAL CARE SYNDROMES

HLH is not a single disease but a final common pathway of toxic immune activation. The syndrome is characterized by uncontrolled activation of T cells and macrophages, excessive cytokine production, impaired immune regulation, tissue infiltration, cytopenias, coagulopathy, and organ injury. In the pediatric ICU (PICU), the presentation is often dynamic rather than complete at onset. Fever and hyperferritinemia may precede worsening cytopenias, hypofibrinogenemia, hepatic dysfunction, shock, respiratory failure, encephalopathy, or renal dysfunction. Because each feature may also occur in sepsis, malignancy, liver failure, or inflammatory disease, clinicians should look for evolving patterns rather than a single diagnostic marker.

A practical bedside trigger for suspicion is the combination of persistent fever, falling blood counts, and high or rapidly rising ferritin. This pattern is not diagnostic by itself, but it should prompt HLH‐focused evaluation in a deteriorating child. Additional clues include splenomegaly, hepatic dysfunction, hypofibrinogenemia, hypertriglyceridemia, elevated soluble IL‐2 receptor, low or absent natural killer (NK) cell activity, hemophagocytosis, neurologic involvement, and inflammatory deterioration out of proportion to the apparent infection burden.^3^

Sepsis and HLH may coexist. In one PICU study of children who died from severe sepsis, all patients had fever and pancytopenia, 82.9% had hepatosplenomegaly, 52.9% had ferritin levels greater than 500 ng/mL, and 35.7% fulfilled at least five of six available HLH‐2004 diagnostic criteria. The authors concluded that there is significant clinical and laboratory overlap between HLH and severe sepsis and that HLH‐2004 criteria should be applied cautiously in this setting.^6^ This caution is particularly important in immunocompromised children, children with malignancy, transplant recipients, children with EBV infection, and patients receiving immune effector cell therapy.

In such cases, the therapeutic question is not whether to treat infection or inflammation, but how to treat both safely. Broad antimicrobial coverage, source control, hemodynamic support, respiratory support, and renal support should proceed in parallel with early collaboration involving hematology, immunology, infectious diseases, rheumatology, nephrology, and critical care.

## DIAGNOSTIC FRAMEWORK FOR THE INTENSIVIST

The HLH‐2004 criteria remain the most widely recognized diagnostic framework in children. They include fever, splenomegaly, cytopenias, hypertriglyceridemia and/or hypofibrinogenemia, hemophagocytosis, low or absent NK cell activity, ferritin elevation, and elevated soluble IL‐2 receptor. Meeting five of eight criteria supports the diagnosis when a molecular diagnosis is not already established.^2^ However, these criteria should be interpreted carefully in the PICU. Many critically ill children with severe sepsis, liver dysfunction, disseminated intravascular coagulation, malignancy, or transfusion exposure may satisfy several criteria without having a classic HLH mechanism.^6^

The diagnostic goal is therefore broader than counting criteria. A useful PICU evaluation asks five practical questions. First, is the child demonstrating progressive hyperinflammation despite appropriate resuscitation and antimicrobial therapy? Second, is there a plausible trigger such as EBV, malignancy, rheumatologic disease, immune effector cell therapy, transplant, or an IEI? Third, is the organ‐failure pattern compatible with HLH or MAS? Fourth, is there a dominant inflammatory pathway supported by the phenotype or by available biomarkers—for example, CXCL9 or interferon‐gamma activity, IL‐18/IL‐1‐family biology, or a clinically defined IL‐6‐dominant cytokine release syndrome—that may inform targeted therapy? Fifth, is the child sick enough that treatment should begin before confirmatory or specialized tests return? Pediatric biomarker studies show that interferon‐gamma‐regulated chemokines, particularly CXCL9, are enriched in HLH compared with sepsis, while IL‐18 and related biomarker patterns may help distinguish systemic JIA‐MAS from other hyperferritinemic syndromes.^7^, ^8^ These signals are supportive and overlapping rather than stand‐alone diagnostic or drug‐selection thresholds.

Initial evaluation should include complete blood count with differential, ferritin, fibrinogen, triglycerides, liver enzymes, bilirubin, coagulation studies, lactate dehydrogenase, D‐dimer, inflammatory markers, soluble IL‐2 receptor when available, NK cell function and degranulation studies when feasible, and infectious evaluation including EBV, cytomegalovirus, adenovirus, herpesviruses, blood cultures, and other tests guided by exposure and immune status. Bone marrow evaluation may identify hemophagocytosis, malignancy, or infection, but absence of hemophagocytosis does not exclude HLH and should not delay treatment in a rapidly deteriorating child.^1^

Genetic testing should be considered early, particularly in infants, recurrent HLH, central nervous system disease, consanguinity, family history, severe EBV susceptibility, unusual infections, or refractory disease. Importantly, targeted familial HLH gene panels may be insufficient because HLH can be the presenting feature of broader IEIs. A systematic review of pediatric HLH associated with IEIs beyond classic familial HLH identified 46 different IEIs and found that HLH preceded the immunodeficiency diagnosis in 75% of cases.^9^ Broader immune and genomic evaluation may identify therapeutic opportunities and clarify whether HSCT is needed.

## ICU PHENOTYPES OF HLH AND CYTOKINE STORM

A phenotype‐based approach is useful because children with similar ferritin levels or HLH‐2004 criteria may require different therapies. Cytokine and chemokine profiles can strengthen phenotype assignment but overlap substantially between syndromes. Familial or primary HLH should be considered in infants and children with recurrent disease, central nervous system involvement, or known or suspected defects in cytotoxic lymphocyte function. Interferon‐gamma pathway activation is prominent in HLH; CXCL9 is a practical surrogate for interferon‐gamma bioactivity, and soluble IL‐2 receptor reflects T‐cell activation.^7^, ^10^, ^11^ These patients often need urgent inflammation control as a bridge to HSCT.

EBV‐associated HLH is a major pediatric phenotype and is particularly prominent in East Asia, but it should not be universally described as the most common form of childhood HLH.^12^ EBV‐HLH ranges from acute infection‐triggered hyperinflammation to chronic active EBV disease (CAEBV) and EBV‐associated T‐cell or NK‐cell lymphoproliferation. Actionable signals include serial EBV DNA burden, the infected‐cell lineage when measurable, and evidence of T/NK‐cell and interferon‐gamma pathway activation; CXCL9 and soluble IL‐2 receptor may support that biology but are not specific for EBV‐HLH. The infected‐cell lineage matters because B‐cell‐predominant disease may respond to rituximab‐containing therapy, whereas T/NK‐cell disease requires different disease‐control strategies.^12^, ^13^ In refractory or relapsed EBV‐HLH—especially CAEBV, persistent T/NK‐cell disease, or suspected genetic susceptibility—early transplant consultation and prompt planning for allogeneic HSCT are essential. Targeted agents such as ruxolitinib or emapalumab may help control hyperinflammation, but should be viewed as bridges rather than substitutes for definitive transplantation when it is indicated.^14^, ^15^, ^16^, ^17^

Malignancy‐associated HLH may accompany lymphoma, leukemia, histiocytic disease, or treatment‐related immune dysfunction. The inflammatory profile is usually mixed rather than defined by one cytokine; interferon‐gamma/CXCL9 and IL‐6 can be elevated in HLH broadly, but no validated cytokine pattern distinguishes malignancy‐associated HLH sufficiently to direct therapy in isolation.^7^, ^18^ The highest‐value “precision” variable is therefore rapid identification of the malignancy and coordination of inflammation control with cancer‐directed therapy. In a pediatric single‐center cohort of 44 patients, lymphoma was the most common associated malignancy, followed by acute leukemia and Langerhans cell histiocytosis; poor prognostic factors included ferritin levels greater than 5000 ng/mL, age older than 10 years, lactate dehydrogenase greater than 500 U/L, and failure to achieve complete remission.^19^

In this phenotype, treating HLH without identifying and treating the malignancy risks achieving only transient inflammatory control without durable disease control, while uncontrolled hyperinflammation may prevent safe initiation of cancer‐directed therapy. Adult malignancy‐associated HLH literature similarly emphasizes persistent tumor antigen stimulation, infection, chemotherapy, and transplantation as interacting drivers.^20^ Although adult data should not be directly extrapolated to children, they reinforce the need to treat malignancy‐associated HLH as both an inflammatory and oncologic emergency.

Macrophage activation syndrome is most often associated with systemic juvenile idiopathic arthritis, systemic lupus erythematosus, or other autoimmune and autoinflammatory diseases. Its biology can include interferon‐gamma/CXCL9, IL‐18, IL‐1‐family cytokines, IL‐6, and tumor necrosis factor.^8^, ^21^ High IL‐18 and related biomarker patterns can support MAS‐like biology but are not sufficiently specific to define treatment alone. This profile provides a mechanistic rationale for corticosteroids and, when disease is refractory or rapidly progressive, IL‐1 blockade with anakinra; additional therapy should be individualized with rheumatology and hematology input.

Sepsis‐associated hyperinflammation remains one of the most difficult diagnostic categories. Severe infection may produce broad activation of IL‐1, IL‐6, tumor necrosis factor, and interferon pathways, along with cytopenias, coagulopathy, liver dysfunction, hyperferritinemia, and multiple organ dysfunction. In contrast to classic HLH, no single cytokine profile or ferritin value is validated to mandate HLH‐directed therapy in sepsis. Pediatric studies nevertheless show that an interferon‐gamma/CXCL9 signature can help distinguish HLH from sepsis or systemic inflammatory response syndrome at the group level.^7^ At the bedside, antimicrobial therapy, source control, and organ support remain primary while selective, titratable immunomodulation is considered only when hyperinflammation is persistent or disproportionate.

Immune effector cell‐associated cytokine storm, including cytokine release syndrome (CRS) and immune effector cell‐associated neurotoxicity syndrome (ICANS), provides the clearest example in which treatment is driven by a defined clinical syndrome and severity grade rather than a measured cytokine threshold. IL‐6 is a prominent therapeutic target in CRS, supporting tocilizumab‐based treatment according to institutional and consensus protocols.^22^ Persistent fever, hyperferritinemia, cytopenias, hypofibrinogenemia, and organ dysfunction beyond the expected CRS course should prompt evaluation for an immune effector cell‐associated HLH‐like syndrome rather than repeated IL‐6 blockade alone.

IEI‐associated HLH is mechanistically heterogeneous. Cytotoxicity defects frequently converge on interferon‐gamma‐driven inflammation, whereas selected immune‐dysregulatory or autoinflammatory disorders may have prominent IL‐18 or other pathway signals.^5^, ^9^ The practical priority is early broad immunologic and genetic evaluation, combined with infection‐directed therapy and a transplant plan when the molecular diagnosis or clinical course indicates that definitive immune reconstitution is required. Table 1 summarizes these ICU phenotypes, pathway signals, and management priorities.

**TABLE 1:** Pediatric intensive care unit phenotypes of hemophagocytic lymphohistiocytosis and cytokine storm.

Phenotype	Trigger or setting	Dominant or indicative immune signals^*^	Bedside clues	Priority action
Familial or primary HLH	Inherited cytotoxic lymphocyte defects; viral triggers may unmask disease	IFN‐gamma pathway activation; CXCL9/CXCL10/CXCL11; elevated sCD25/T‐cell activation	Infancy/early childhood, recurrent disease, CNS involvement, persistent cytopenias, high ferritin	Urgent HLH control; genetic/immunologic evaluation; infection surveillance; early HSCT planning
EBV‐associated HLH	Primary EBV infection, chronic active EBV, EBV‐susceptibility disorders; B‐, T‐, or NK‐cell infection	Serial EBV DNA and infected‐cell lineage are actionable trigger markers; IFN‐gamma/CXCL9 and sCD25 may be elevated	Persistent fever, hepatosplenomegaly, cytopenias, hepatitis, high or persistent EBV burden, shock/MODS	Etoposide‐based/HLH therapy as indicated; identify lineage; rituximab if B‐cell predominant; early allo‐HSCT planning for refractory/relapsed, CAEBV, or persistent T/NK disease
Malignancy‐associated HLH	Lymphoma, leukemia, histiocytic disease; during chemotherapy or immunosuppression	Mixed cytokine activation; IFN‐gamma/CXCL9 and IL‐6 may be elevated but are not malignancy‐specific	Fever, cytopenias, high ferritin/LDH, hypoalbuminemia; tumor features may be masked	Control hyperinflammation while expediting tissue diagnosis and cancer‐directed therapy; do not treat HLH in isolation
Macrophage activation syndrome	Systemic JIA, SLE, autoinflammatory disease, infection‐triggered flare	IL‐18, IL‐1‐family cytokines, IFN‐gamma/CXCL9, IL‐6; overlapping rather than diagnostic	Sudden deterioration, fever, falling platelets/fibrinogen, rising ferritin, hepatitis, shock	Steroids; consider anakinra for refractory/rapidly progressive MAS; trigger control and rheumatology involvement
Sepsis‐associated hyperinflammation/HLH‐like MODS	Severe bacterial, viral, or fungal infection; immunocompromised host	Broad IL‐1/IL‐6/TNF/interferon activation; no validated cytokine cutoff mandates HLH therapy	Persistent fever despite treatment, hyperferritinemia, cytopenias, coagulopathy, organ failure out of proportion to source	Treat infection and source aggressively; consider selective, titratable immunomodulation only when hyperinflammation persists/disproportionate
Immune effector cell‐associated cytokine storm	CAR T cells, bispecific antibodies, related immune effector therapy	IL‐6 is a prominent therapeutic pathway; treatment is guided primarily by clinical CRS/ICANS grade	Fever, hypotension, hypoxia, capillary leak, neurologic toxicity; persistent HLH‐like laboratory features	Use institutional CRS/ICANS protocols and tocilizumab/steroids when indicated; evaluate persistent disease for HLH‐like syndrome
Inborn error of immunity‐associated HLH	Combined immunodeficiency, immune dysregulation, cytotoxicity defects, EBV susceptibility	Mechanism‐specific; cytotoxicity defects often converge on IFN‐gamma/CXCL9; selected disorders may have prominent IL‐18	Severe/recurrent or unusual infections, family history, early/recurrent HLH, poor response to standard therapy	Broad immunologic/genetic evaluation; tailor prophylaxis/immunomodulation; define HSCT need early

* Pathway signals are supportive, often overlapping, and should not be interpreted as validated diagnostic or drug‐selection thresholds.

Abbreviations: CAEBV, chronic active Epstein‐Barr virus disease; CAR, chimeric antigen receptor; CNS, central nervous system; CRS/ICANS, cytokine release syndrome/ immune effector cell‐associated neurotoxicity syndrome; EBV, Epstein‐Barr virus; HLH, hemophagocytic lymphohistiocytosis; HSCT, hematopoietic stem cell transplantation; IFN, interferon; IL, interleukin; JIA, juvenile idiopathic arthritis; LDH, lactate dehydrogenase; MODS, multiple organ dysfunction syndrome; NK, natural killer; SLE, systemic lupus erythematosus; TNF, tumor necrosis factor.

## PRECISION IMMUNOMODULATION: MATCHING THERAPY TO PHENOTYPE

In the PICU, precision immunomodulation is an iterative bedside process rather than a one‐time drug choice. Four inputs should be reviewed together: trigger/phenotype, pathway signal, ICU drug‐delivery constraints, and the definitive disease‐control plan. Specialized cytokine tests should not delay treatment. CXCL9 can support interferon‐gamma activity, IL‐18 can support MAS‐like biology, and EBV DNA burden and infected‐cell lineage can refine EBV‐HLH management; however, no universally validated cutoff for CXCL9, interferon‐gamma, IL‐18, or any other cytokine mandates a specific agent.^7^, ^8^, ^10^, ^11^ Table 2 translates these signals into practical treatment considerations and 24–72‐h reassessment points.

**TABLE 2:** Bedside biomarker‐phenotype‐therapy matching for pediatric hemophagocytic lymphohistiocytosis and cytokine storm in the pediatric intensive care unit

Phenotype or pathway signal	Actionable bedside interpretation	Therapy to consider	ICU caveats and reassessment
Primary/refractory HLH; IFN‐gamma‐dominant biology supported by CXCL9 when available	Strongest evidence for direct IFN‐gamma blockade; no numeric CXCL9 threshold is required	Emapalumab with dexamethasone/HLH backbone; bridge to HSCT^10^, ^24^, ^25^, ^26^	Screen for intracellular/fungal/mycobacterial/viral infection; reassess disease activity and organ support within 24–72 h
Refractory/relapsed EBV‐HLH; persistent EBV DNA, especially T/NK‐cell disease or CAEBV	Trigger remains active despite initial HLH control; definitive therapy may require immune reconstitution	Etoposide‐based therapy as indicated; select ruxolitinib or emapalumab; rituximab if B‐cell predominant; prompt allo‐HSCT planning^13^, ^14^, ^15^, ^17^, ^27^, ^31^	Do not allow transient biomarker improvement to delay transplant evaluation; trend EBV DNA and organ dysfunction
Broad multi‐cytokine/refractory HLH with enteral access	JAK1/2 blockade can suppress several inflammatory pathways when no single target dominates	Ruxolitinib^16^, ^28^	Enteral absorption may be unreliable in shock/ileus; monitor cytopenias, liver function, infection and drug interactions; early response expected in responders
MAS/secondary HLH‐like phenotype; IL‐18/IL‐1‐family biology supported by clinical context	Short‐acting, titratable IL‐1 blockade is attractive when diagnosis is evolving	Corticosteroids plus/minus anakinra; IV route in selected critically ill patients^21^, ^29^, ^30^	IL‐1 level is not required; IL‐18 is supportive, not a treatment cutoff; adjust for renal function and monitor infection
Immune effector cell CRS; IL‐6 pathway clinically dominant	Treatment is based on syndrome and ASTCT/institutional grade, not measured IL‐6	Tocilizumab plus/minus corticosteroids per protocol^22^	Persistent hyperferritinemia, cytopenias, hypofibrinogenemia, or organ failure should prompt evaluation for HLH‐like syndrome
Familial/aggressive HLH requiring rapid cytotoxic control	Activated T‐cell/macrophage disease remains a core therapeutic target	Corticosteroids and etoposide^1^, ^2^	Hematology‐led dosing with hepatic/renal dysfunction and cytopenias; infection surveillance; bridge to definitive therapy
Fulminant hyperferritinemic sepsis/MODS, MAS or secondary HLH‐like syndrome in selected cases	Temporary removal of circulating mediators may provide a non‐cytotoxic bridge	Therapeutic plasma exchange^32^	Evidence remains limited; vascular access/hemodynamic feasibility and removal of therapeutic drugs/antibodies must be considered
AKI, fluid overload, severe acidosis or electrolyte derangement during cytokine storm	Primary role is organ support; mediator clearance is secondary and membrane‐dependent	Continuous renal replacement therapy^1^	Not definitive immunotherapy; adjust antimicrobial/biologic dosing and coordinate timing
Fulminant cytokine storm with rescue‐level mediator burden after disease‐directed therapy started	Case‐level evidence only for extracorporeal adsorption	Hemoadsorption/cytokine adsorption^33^	May remove therapeutic drugs; should not delay trigger‐ or HLH‐directed therapy
Potentially reversible refractory cardiopulmonary failure	Organ rescue may create time for trigger‐directed and immune‐directed therapy to work	ECMO in selected cases^1^	Bleeding/thrombosis, infection, anticoagulation and reversibility must be assessed; disease‐directed therapy must continue

Abbreviations: AKI, acute kidney injury; ASTCT, American Society for Transplantation and Cellular Therapy; CAEBV, chronic active Epstein‐Barr virus disease; CRS, cytokine release syndrome; EBV, Epstein‐Barr virus; ECMO, extracorporeal membrane oxygenation; HLH, hemophagocytic lymphohistiocytosis; HSCT, hematopoietic stem cell transplantation; ICU, intensive care unit; IFN, interferon; IL, interleukin; JAK, Janus kinase; MAS, macrophage activation syndrome; MODS, multiple organ dysfunction syndrome; NK, natural killer. Biomarker results are supportive and should not delay treatment of an unstable child.

Targeted and adjunctive therapies should complement, not replace, corticosteroids, etoposide when indicated, antimicrobial therapy, trigger control, and organ support. Other specialist‐directed salvage therapies, including alemtuzumab, remain outside the core ICU framework and are not reviewed in detail.^23^ The most useful ICU question is not “Which cytokine is high?” but “Which pathway is most plausible, which treatment can be delivered safely now, and what definitive therapy must this treatment bridge to?”

## ICU‐FOCUSED TARGETED IMMUNOMODULATION

Corticosteroids remain a rapid, widely available initial anti‐inflammatory treatment in many unstable HLH or MAS phenotypes. Etoposide remains central for familial HLH and severe, persistent, relapsing, or selected EBV‐ and malignancy‐associated disease in which activated T‐cell/macrophage inflammation requires urgent control.^1^, ^2^ In critical illness, dosing and timing should be coordinated with hematology‐oncology when hepatic dysfunction, renal dysfunction, profound cytopenias, or active infection increase toxicity risk.

Emapalumab is most strongly supported in primary HLH that is refractory, recurrent, progressive, or intolerant of conventional therapy.^10^, ^24^, ^25^, ^26^ CXCL9 is produced downstream of interferon‐gamma receptor activation and can serve as a practical marker of interferon‐gamma activity; pharmacometrics work in primary HLH supports using clinical disease activity together with interferon‐gamma/CXCL9 biology for treatment adaptation rather than relying on a single static value.^11^ In refractory EBV‐HLH, published pediatric experience is limited; emapalumab may be used as a bridge to disease control and allogeneic transplantation in selected patients.^14^, ^15^, ^27^ Infection surveillance, including fungal, mycobacterial, and viral risk assessment, remains essential.

Ruxolitinib inhibits JAK1/2 and therefore suppresses signaling from multiple cytokine pathways rather than a single cytokine pathway. This makes it most attractive when refractory HLH has broad cytokine activation, when EBV‐HLH is not adequately controlled by initial therapy, or when a rapidly acting non‐cytotoxic bridge is desired, and enteral absorption is reliable. In a pediatric response‐based study of ruxolitinib as frontline monotherapy in newly diagnosed HLH, the overall response rate after 4 weeks of monotherapy was 69.2%, with first responses within three days among responders; EBV‐HLH showed the highest response rate at 87.5%, although additional intensive therapy was still required in many patients, and these data should be extrapolated to refractory disease with caution.^16^, ^28^ Cytopenias, infection risk, drug interactions, hepatic function, and unreliable enteral absorption during shock should be reassessed early.

Anakinra is most useful when the phenotype is MAS or secondary HLH‐like hyperinflammation with a plausible IL‐1/IL‐18 axis, particularly when diagnostic uncertainty makes a short‐acting and titratable agent preferable.^8^, ^21^, ^29^ A measured IL‐1 concentration is not required to justify therapy, and IL‐18 is supportive rather than a validated treatment threshold. Intravenous administration may be preferable in selected critically ill children with edema, thrombocytopenia, poor subcutaneous perfusion, or a need for high doses.^29^, ^30^ Anakinra should not be used as sole definitive therapy for familial HLH, aggressive EBV‐HLH, or disease requiring transplantation.

Tocilizumab is primarily a syndrome‐directed rather than biomarker‐threshold‐directed therapy. In children receiving chimeric antigen receptor (CAR) T cells or related immune effector therapies, tocilizumab and corticosteroids should follow institutional CRS/ICANS protocols and clinical severity grading.^22^ Persistent HLH‐like features after expected CRS treatment should trigger a broader HLH evaluation and reassessment of the inflammatory target rather than automatic repetition of IL‐6 blockade.

## DEFINITIVE DISEASE CONTROL AND TRANSPLANT PLANNING

Transplant planning is part of precision treatment, not a downstream consideration after every targeted option has failed. Primary/familial HLH often requires HSCT for cure.^2^, ^5^ For refractory or relapsed EBV‐HLH, particularly CAEBV or persistent EBV‐positive T/NK‐cell disease, transplant consultation should occur early, and allogeneic HSCT should be planned promptly once disease control permits.^17^, ^31^ Published pediatric cases and series support using targeted agents as bridges to transplantation, not as substitutes when the underlying biology requires immune reconstitution.^14^ This distinction is especially important in the PICU, where temporary improvement in fever or ferritin can otherwise create false reassurance.

## EXTRACORPOREAL AND ADJUNCTIVE STRATEGIES

Extracorporeal strategies are ICU bridge therapies rather than definitive immunomodulation. Continuous renal replacement therapy should be used for conventional indications such as acute kidney injury, severe fluid overload, metabolic acidosis, or electrolyte derangements; any cytokine clearance is secondary and membrane‐dependent. Therapeutic plasma exchange can be considered in selected hyperferritinemic sepsis/multiple organ dysfunction syndrome, MAS, or secondary HLH‐like syndromes when fulminant inflammation coexists with organ failure, and clinicians seek a temporizing, non‐cytotoxic bridge, but pediatric evidence remains limited.^32^ Hemoadsorption has only case‐level evidence in HLH and should not delay disease‐directed treatment.^33^

Extracorporeal membrane oxygenation (ECMO) may be appropriate for selected children with potentially reversible refractory cardiopulmonary failure while trigger‐directed and HLH‐directed therapy continues. Risks of bleeding, thrombosis, infection, anticoagulation requirements, and the likelihood of reversible inflammatory injury must be weighed before cannulation. Plasma exchange, hemoadsorption, and continuous renal replacement therapy can alter exposure to antimicrobials and biologics; timing of emapalumab, anakinra, and other agents should therefore be coordinated with the pharmacy, nephrology, hematology, and ECMO teams.

## MONITORING, SAFETY, AND RESPONSE ASSESSMENT

Precision immunomodulation is only as safe as the monitoring system around it. Response should be assessed serially rather than by a single laboratory value. Important markers include fever curve, vasoactive requirement, oxygenation, neurologic status, ferritin trend, fibrinogen, cytopenias, liver injury, lactate dehydrogenase, soluble IL‐2 receptor when available, viral loads, and organ dysfunction trajectory. Pathway biomarkers should be trended only when they are clinically available and actionable: CXCL9 (with or without interferon‐gamma) can support the monitoring of response to interferon‐gamma‐directed therapy, IL‐18 can support MAS‐like biology, and serial EBV DNA monitoring is central in EBV‐HLH.

A practical PICU monitoring and safety checklist is provided in Table 3.

**TABLE 3:** Pediatric intensive care unit monitoring and safety checklist during precision immunomodulation for hemophagocytic lymphohistiocytosis and cytokine storm

Domain	What to monitor	Why it matters	Practical action
Hyperinflammation	Fever curve, ferritin, fibrinogen, triglycerides, cytopenias, liver enzymes, LDH, D‐dimer, soluble IL‐2 receptor; CXCL9 ± IFN‐gamma when IFN‐gamma blockade is being considered; IL‐18 when MAS biology is suspected	Defines overall inflammatory trajectory and may support pathway‐directed treatment when specialized tests are available	Review daily trends; do not wait for cytokine tests to treat an unstable child; interpret pathway markers with phenotype and organ response
Hemodynamics	Vasoactive‐inotropic support, lactate, capillary refill, urine output, echocardiography when myocardial dysfunction is suspected	HLH/cytokine storm may cause distributive shock, myocardial dysfunction, capillary leak, or mixed shock	Escalate vasoactive support while treating inflammation and infection; consider cardiology/ECMO consultation for refractory shock
Respiratory failure	Oxygen requirement, oxygenation index, ventilator settings, chest imaging, fluid balance	ARDS and capillary leak may accompany cytokine storm and worsen outcomes	Apply lung‐protective ventilation, optimize fluid strategy, consider prone positioning or ECMO evaluation when appropriate
Neurologic involvement	Mental status, seizures, focal deficits, neuroimaging, cerebrospinal fluid studies when safe, ICANS grading after immune effector therapy	CNS HLH, encephalopathy, seizures, or ICANS may change therapy and prognosis	Initiate seizure precautions, neurology involvement, CNS‐directed evaluation, and cellular‐therapy toxicity protocols when relevant
Infection surveillance	Blood/urine/respiratory cultures, EBV, cytomegalovirus, adenovirus, herpes simplex virus, varicella zoster virus testing as appropriate, fungal and mycobacterial evaluation in high‐risk patients	Targeted immunomodulation can worsen occult infection or viral/fungal reactivation	Maintain broad antimicrobial coverage when infection is possible; trend viral loads; involve infectious diseases early
Malignancy evaluation	Peripheral smear, LDH, uric acid, imaging, marrow evaluation, flow cytometry, oncology consultation	Malignancy‐associated HLH may be the presenting feature of lymphoma, leukemia, or histiocytic disease	Do not treat HLH in isolation; expedite tissue diagnosis and cancer‐directed therapy when suspected
Genetic and immunologic evaluation	Familial HLH genes, degranulation or natural killer cell function when available, broader inborn error of immunity testing, immunoglobulins, lymphocyte subsets	HLH may reveal primary HLH or other inborn errors of immunity	Send testing early; do not wait for results before treating unstable patients; use results to guide HSCT planning
Drug toxicity	Cytopenias, transaminases, bilirubin, creatinine, blood pressure, neurologic changes, drug levels when relevant	Etoposide, cyclosporine, JAK inhibitors, biologics, and antimicrobials may cause overlapping toxicities	Adjust dosing for organ dysfunction; coordinate pharmacy, hematology, nephrology, and infectious diseases
Viral/fungal reactivation	Cytomegalovirus, EBV, adenovirus, fungal markers/cultures when appropriate, tuberculosis/mycobacterial risk before interferon‐gamma blockade	Interferon‐gamma blockade and combination immunosuppression may increase opportunistic infection risk	Screen before and during therapy when feasible; consider prophylaxis based on risk and institutional practice
Extracorporeal therapy interactions	Continuous renal replacement therapy settings, plasma exchange schedule, hemoadsorption timing, biologic dosing timing	Extracorporeal therapies may remove cytokines but may also alter drug or antibody exposure	Coordinate timing of emapalumab, anakinra, antimicrobials, and other agents with nephrology/pharmacy
Response and escalation	Fever resolution, organ‐support trajectory, ferritin/fibrinogen/cytopenia trends, EBV DNA when relevant, CXCL9/IFN‐gamma or IL‐18 when clinically available	Nonresponse may signal wrong phenotype/pathway, uncontrolled trigger, inadequate drug exposure, or need for definitive therapy	Define reassessment at 24–72 h; redirect targeted therapy, intensify etoposide‐based therapy when indicated, advance HSCT planning, or add extracorporeal support as appropriate
Family communication	Diagnostic uncertainty, evolving treatment plan, infection risk, transplant possibility, prognosis	HLH/cytokine storm management changes rapidly and may involve high‐risk therapies	Provide frequent updates; explain that therapy is often adjusted based on response and evolving diagnostic information

Abbreviations: ARDS, acute respiratory distress syndrome; CNS, central nervous system; EBV, Epstein‐Barr virus; ECMO, extracorporeal membrane oxygenation; HLH, hemophagocytic lymphohistiocytosis; HSCT, hematopoietic stem cell transplantation; ICANS, immune effector cell‐associated neurotoxicity syndrome; IFN, interferon; IL, interleukin; MAS, macrophage activation syndrome; JAK, Janus kinase; LDH, lactate dehydrogenase.

Infection surveillance is central. Children with HLH are vulnerable to infection because of the underlying disease, immune dysregulation, cytopenias, mucosal injury, central venous catheters, organ failure, and immunomodulatory treatment. EBV, cytomegalovirus, adenovirus, herpes simplex virus, varicella zoster virus, fungal pathogens, and mycobacteria should be considered based on phenotype and therapy. Interferon‐gamma blockade, JAK inhibition, etoposide, alemtuzumab, and combination immunosuppression all increase the need for structured surveillance and prophylaxis decisions.^10^, ^15^

Nonresponse should prompt reassessment rather than automatic repetition of the same intervention. Potential explanations include uncontrolled infection, occult malignancy, unrecognized IEI, inadequate drug delivery, impaired enteral absorption, drug removal by extracorporeal therapy, an incorrect inflammatory target, progression of EBV‐positive T/NK‐cell disease, or irreversible organ injury. Reassessment at 24–72 h is practical in the PICU because clinically meaningful trends in fever, vasoactive support, ferritin, fibrinogen, cytopenias, viral load, pathway biomarkers when available, and organ dysfunction often emerge over that interval.^1^ A phenotype or pathway mismatch should trigger treatment redirection and renewed attention to definitive disease control, including HSCT when indicated.

Family communication should be proactive. HLH and cytokine storm management often involves uncertainty, rapidly changing information, and therapies with significant risk. Families should understand that clinicians may begin treatment before all test results return, adjust therapy based on response, and involve multiple teams because the syndrome spans critical care, hematology‐oncology, immunology, infectious diseases, rheumatology, nephrology, neurology, transplant, and cellular therapy.

## GLOBAL ACCESS AND IMPLEMENTATION CHALLENGES

Precision immunomodulation raises important equity questions. Molecular diagnostics, soluble cytokine testing, NK cell functional testing, broad viral surveillance, emapalumab, JAK inhibitors, anakinra, hemoadsorption devices, and transplant services are not equally available across institutions or countries. Even within high‐resource settings, turnaround time for genetic testing or specialized immune assays may exceed the therapeutic window of a critically ill child.

A practical implementation strategy should therefore include both high‐resource and resource‐adapted pathways. At minimum, institutions should develop a bedside hyperinflammation screen using readily available tests such as complete blood count, ferritin, fibrinogen, triglycerides, liver enzymes, coagulation studies, and infection evaluation. Pathways should define when to call hematology, immunology, infectious diseases, rheumatology, and critical care specialists; when to order specialized testing; and when to begin empiric immunomodulation despite incomplete data. Where targeted biologics are unavailable, early recognition, antimicrobial therapy, steroids, etoposide when indicated, intravenous immune globulin, plasma exchange in selected contexts, and transfer pathways may still improve care.

## LIMITATIONS

This is a narrative review rather than a systematic review, and the selection of cited evidence reflects clinical relevance rather than a prespecified protocol. Much of the evidence for targeted immunomodulation in refractory pediatric HLH derives from small, frequently single‐center, retrospective cohorts and case series, and some inferences are extrapolated from adult or non‐critical‐care populations. These constraints reflect the rarity and biologic heterogeneity of the syndrome and underscore the need for multicenter, prospective, phenotype‐stratified studies to define optimal agent selection, sequencing, and timing.

## CONCLUSION

Refractory HLH and cytokine storm in the PICU require more than application of diagnostic criteria or protocolized escalation. These syndromes should be understood as dynamic hyperinflammatory organ‐failure phenotypes arising from diverse mechanisms. Precision immunomodulation offers a path toward more individualized care, but it must be integrated with antimicrobial therapy, trigger control, organ support, infection surveillance, and transplant planning. For the pediatric intensivist, the goal is not only to suppress inflammation, but to identify the right child, the right pathway, the right timing, and the safest bridge to durable disease control.

## CONFLICT OF INTEREST

The authors declare no conflict of interest.
